# 血清细胞角蛋白19在预测进展期非小细胞肺癌患者化疗疗效及预后中的临床意义

**DOI:** 10.3779/j.issn.1009-3419.2010.10.05

**Published:** 2010-10-20

**Authors:** 崇安 许, 佳丽 刘, 丽丽 邢, 殊 刘

**Affiliations:** 110032 沈阳，中国医科大学附属第四医院肿瘤内科 Department of Oncology Medicine, the Fourth Affiliated Hospital of China Medical University, Shenyang 110032, China

**Keywords:** 细胞角蛋白19片段, 肺肿瘤, 化疗疗效, 影像学缓解, 预测因素, 预后因素, CYFRA21-1, Lung neoplasms, Chemotherapy efficacy, Radiological response, Predictor factors, Prognostic factors

## Abstract

**背景与目的:**

由于RECIST（Response Evaluation Criteria in Solid Tumors, RECIST）标准不能对存活肿瘤组织进行检测，也不能对所有无法测量病灶的非小细胞肺癌（non-small cell lung cancer, NSCLC）患者的疗效进行准确评估，本研究通过检测进展期NSCLC患者化疗前后血清细胞角蛋白19片段（cytokeratin 19 fragment, CYFRA21-1）表达水平的变化以评价其在预测进展期NSCLC患者化疗疗效及预后中的临床价值。

**方法:**

采用全自动生化分析仪电化学发光免疫法检测112例初治的NSCLC患者化疗前和化疗2周期后血清CYFRA21-1表达水平的变化，应用受试者特征工作曲线（receiver operating characteristics curve, ROC）评价血清CYFRA21-1反应在诊断影像学缓解（objective response, OR）中的效能及其与预后的相关性。

**结果:**

经一线铂类为基础的两药联合方案化疗2周期后，血清CYFRA21-1水平较化疗前基线水平明显下降。80例可评价影像学和血清学疗效的患者中，26.3%（21/80）的患者达到影像学OR。化疗2周期后40.0%（32/80）的患者血清CYFRA21-1水平下降≥60%（血清CYFRA21-1反应）。血清CYFRA21-1反应与影像学OR之间有明显的统计学相关性（*P* < 0.001）。所有患者的中位生存期为9.9个月，血清CYFRA21-1水平下降≥60%患者的生存期明显长于CYFRA21-1水平下降 < 60%的患者（12.3个月*vs* 8.9个月，*P* < 0.001）。单因素分析结果显示，血清CYFRA21-1基线水平、CYFRA21-1反应、PS评分及影像学OR是影响生存期的重要预后因素。*Cox*多因素生存分析证实，仅血清CYFRA21-1基线水平、CYFRA21-1反应及PS评分是影响预后生存期的独立因素，而OR则与预后无关。

**结论:**

血清CYFRA21-1水平可敏感地反映影像学肿瘤体积大小的变化，可能是评价进展期NSCLC患者化疗疗效的替代指标，同时也是预测预后生存期的可信指标。

## Abstract (en)

**Background and objective:**

RECIST (Response Evaluation Criteria in Solid Tumors) criteria could not be used to detect viable tumor tissue and is not an accurate tool for evaluation of objective response (OR) in non-small cell lung cancer (NSCLC) patients without measurable lesions. The aim of this study is to detect the pre- and post-chemotherapy serum cytokeratin 19 fragment (CYFRA21-1) expression levels in advanced NSCLC patients to evaluate the clinical value of CYFRA21-1 in the prediction of chemotherapy response and prognosis in NSCLC patients.

**Methods:**

A automatic electrochemiluminescence immunoassay analyzer was applied to detect the pre- and post-chemotherapy serum CYFRA21-1 expression levels in 112 cases of initial treatment patients with NSCLC. Application of receiver operating characteristics curve (ROC) curve in evaluation the significance of serum CYFRA21-1 response in the diagnosis of OR and its correlation with prognosis.

**Results:**

After 2 cycles of platinum-based combined chemotherapy, post-chemotherapy serum CYFRA21-1 significantly decreased compared with baseline levels. 80 patients were evaluable for radiological and serological efficacy, and 26.3% (21/80) patients achieved radiological OR. The decrease ratio of post-chemotherapy serum CYFRA21-1 with CYFRA21-1 response was 40.0% (32/80). There was significant correlation between serum CYFRA21-1 response and OR (*P* < 0.001). The median survival time of all patients was 9.9 months; the survival of patients with CYFRA21-1 response were significantly longer than those without CYFRA21-1 responders (12.3 months *vs* 8.9 months, *P* < 0.001). Univariate survival analysis showed that PS score, OR, baseline serum CYFRA21-1 level and CYFRA21-1 response were important prognostic factors. *Cox* multivariate survival analysis confirmed that only the PS score, serum CYFRA21-1 baseline levels and CYFRA21-1 response were independent prognostic factors of NSCLC patients; OR was not an independent prognostic factor.

**Conclusion:**

Serum CYFRA21-1 level can be sensitive to reflect changes in tumor volume, which may be a reliable substitution index for evaluation the chemotherapy efficacy in advanced NSCLC patients and a good indicator for prediction the prognosis of survival.

由于大多数非小细胞肺癌（non-small cell lung cancer, NSCLC）患者确诊时已为失去手术机会的进展期，故化疗成为其主要治疗手段。目前的证据^[1]^表明，含铂二药联合方案化疗可有效地提高进展期NSCLC患者的生活质量，延长其生存期，并已成为进展期NSCLC的标准治疗方案。但是相关数据显示，多数进展期NSCLC患者仍不能从化疗中获益^[2]^，化疗仅对约50%初治患者有效，且多为部分有效^[3]^。由于化疗有效可使患者获益，而反映化疗有效的影像学缓解或客观有效率（objective response, OR）与生存相关，故OR已成为评价化疗疗效的替代指标^[4]^，临床决策患者是否继续化疗亦是依据影像学是否达到OR而制定的，它被视为临床受益情况的指标。但是仅以影像学可测量病灶大小的变化作为判定NSCLC化疗疗效的唯一标准，不但有其自身的局限性，也不能完全反映化疗的真实疗效^[5]^，而且化疗的OR也不能完全预测NSCLC患者的生存期^[6]^。因此，寻找评价进展期NSCLC患者化疗疗效和预测生存期的简便易行的方法具有重要的临床意义。细胞角蛋白19片段（cytokeratin 21 fragment, CYFR A21-1）是NSCLC的特异性肿瘤标志物，目前已被证实具有预测NSCLC患者化疗疗效及预后的潜力^[7]^，但是它作为评定化疗疗效和判定预后的替代指标的研究迄今仍未广泛开展。本研究旨在通过比较进展期NSCLC患者化疗前后血清CYFR A21-1水平的变化作为评价进展期NSCLC患者化疗疗效的替代指标及其预测预后生存期的临床价值。

## 1 材料与方法

### 1.1 研究对象

所有病例均来源于2005年1月-2010年1月中国医科大学附属第四医院住院患者，凡未经治疗并接受一线联合化疗的进展期NSCLC患者均可纳入本研究。入组标准：①能够承受至少2个周期一线含铂二药方案联合化疗，并且经细胞学或组织病理学证实的初治NSCLC患者；②不可切除性Ⅲ期-Ⅳ期NSCLC患者；③ECOG PS评分为0分-2分；④具有可测量病灶；⑤肺、心、肝、肾功能和造血功能正常，无严重的合并症。排除标准：①脑转移患者；②术后复发患者。

### 1.2 方法

血液标本在患者知情同意的情况下，于化疗前及化疗2个周期结束后采集。应用全自动生化分析仪电化学发光免疫法检测血清CYFRA21-1水平，血清CYFRA21-1的检测严格依据试剂生产厂家的说明书实施，结果以ng/mL表示。血清CYFRA21-1筛选的临界值为3.2 ng/mL。化疗前和化疗2周期结束后两组患者血清CYFRA21-1至少有1组超过正常阈值（3.2 ng/mL）即为可评价病例。

### 1.3 疗效评定

根据RECIST标准（Response Evaluation Criteria in Solid Tumors, RECIST）评定疗效，分为完全缓解（complete response, CR）、部分缓解（partial response, PR）、疾病稳定（stable disease, SD）和疾病进展（progressive disease, PD）。化疗2个周期后评定疗效，将CR+PR定义为影像学有效或OR，SD+PD视为影像学无效。为评价影像学疗效，需于化疗前和每2个化疗周期后行胸部和上腹部CT检查。

### 1.4 生存期计算

生存期从化疗开始计算，直至死亡或最后一次随访，随访采用回院治疗、复查、走访、随访信或电话询问等方式，随访截止时间为2010年5月。

### 1.5 统计学方法

计量资料（数值变量）采用非参数检验，化疗前和化疗2周期后血清CYFRA21-1水平变化采用秩和检验（*Wilcoxon signed-rank* test，符号秩和检验），*Spearman*相关系数（秩相关系数）评价变量之间关系。计数资料（分类变量）之间的关联采用χ^2^检验。采用受试者特征工作曲线（receiver operating characteristics curve, ROC）评价CYFRA21-1表达水平在预测治疗效果中的效能。应用Youden指数指示CYFRA21-1最佳下降阈值以判定OR。采用单因素生存分析（*Kaplan-Meier*法，*Log-rank*检验）评价不同预后因素对患者生存期的影响。单因素分析中对生存期影响显著（*P* < 0.05）的因素纳入多因素*Cox*回归分析模型进行分析。以*P* < 0.05为差异具有统计学意义，所有检验和*P*值均为双侧。

## 2 结果

### 2.1 患者临床特征

112例未经治疗的进展期NSCLC患者入组本研究。患者的基线特征如下：男性77例（68.8%），中位年龄64岁（31岁-84岁）。56例（50.0%）患者组织学亚型为腺癌，64例（57.1%）为Ⅳ期NSCLC。105例（93.8%）患者ECOG PS评分在0分-1分之间，36例（32.1%）患者白细胞计数（white blood cell, WBC） > 10 000/mm^3^。所有患者均接受了2个-6个周期化疗（平均4个周期），29.5%（33/112）达影像学OR。化疗前血清CYFRA21-1基线水平的中位值为5.15 ng/mL（1.53 ng/mL-95.62 ng/mL），详见表 1。

**1:** 纳入本研究患者的基线特征 Characteristics of included patients

Characteristic	No. of patients (*n*=112)	%
Gender		
Male	77	68.8%
Female	35	31.2%
Age (year)		
Median age (range)	64 (31-84)	
≤65	62	55.4%
>65	50	44.6%
Histology		
Squamous cell carcinoma	37	33.0%
Adenocarcinoma	56	50.0%
NSCLC unspecified	19	20.0%
Clinical stage		
Ⅲ	48	42.9%
Ⅳ	64	57.1%
ECOG PS scores		
0	69	61.6%
1	36	32.1%
2	7	6.2%
WBC count		
Normal (≤10 000/mm^3^)	76	67.9%
Pathologic (>10 000/mm^3^)	36	32.1%
Baseline CYFRA21-1 (ng/mL）		
Median (range)	5.15 (1.53-95.62)	
Normal (≤3.2 ng/mL)	40	35.7%
Elevated (>3.2 ng/mL)	72	64.3%
Chemotherapy efficacy		
CR	1	0.9%
PR	32	28.6%
SD	44	39.3%
PD	35	31.2%
Chemotherapy treatment		
NP	28	25.0%
GP	17	15.2%
TP	9	8.0%
DP	49	43.8%
EP	9	8.0%
Status at follow-up		
Alive	22	19.6%
Dead	90	80.4%
CR: complete response; PR: partial response; SD: stable disease; PD: progressive disease; EP: etoposide+cisplatin; NP: vinorelbine+cisplatin; TP: paclitaxel+cisplatin/ carboplatin; GP: gemcitabine+cisplatin/carboplatin; DP: docetaxel+cisplatin/ carboplatin.		

化疗前血清CYFRA21-1基线水平与年龄（*r*=0.089, *P*=0.353）、性别（*r*=-0.071, *P*=0.458）、组织学类型（*r*=-0.008, *P*=0.933）、疾病分期（*r*=0.029, *P*=0.761）、WBC基线水平（*r*=-0.032, *P*=0.738）及PS评分（*r*=0.099, *P*=0.299）等均无明显相关性。

### 2.2 化疗疗效

所有112例患者中，化疗2周期后有29.5%（33/112）达影像学OR，根据方法学部分介绍的纳入标准，112例患者中有80例（71.4%）血清CYFRA21-1变化水平符合临床疗效评定标准。化疗2周期后，26.3%（21/80）的患者达到影像学OR。

### 2.3 化疗前后血清CYFRA21-1水平变化

可评价的80例患者中，化疗前和化疗2周期后血清CYFRA21-1水平超过正常阈值的患者分别为90.0%（72/80）和63.8%（51/80）。化疗前和化疗2周期后血清CYFRA21-1中位值分别为9.36 ng/mL（2.51 ng/mL-97.15 ng/mL）和4.00 ng/mL（0.73 ng/mL -97.71 ng/mL）；化疗后血清CYFR A21-1水平较化疗前明显下降（*P* < 0.001）。化疗2周期后有60例（75.0%）患者的血清CYFRA21-1水平下降，血清CYFRA21-1下降了27.4%（中位值），其中，治疗有效组和治疗无效组分别下降76.8%和16.7%（*P* < 0.001）。15例治疗有效的患者和14例治疗无效的患者血清CYFRA21-1水平在正常范围内。如图 1所示，x轴表示治疗前血清CYFRA21-1表达水平，y轴表示治疗后血清CYFRA21-1表达水平，该图反映了治疗达到影像学OR所对应的血清标志物水平。若治疗前后血清标志物表达水平无明显差异，则相应的对应点应落在图中所示的直线上。实际上，大多数落点均在该直线以下，提示对于大多数患者而言，化疗可降低血清CYFRA21-1的表达水平。

**1:**
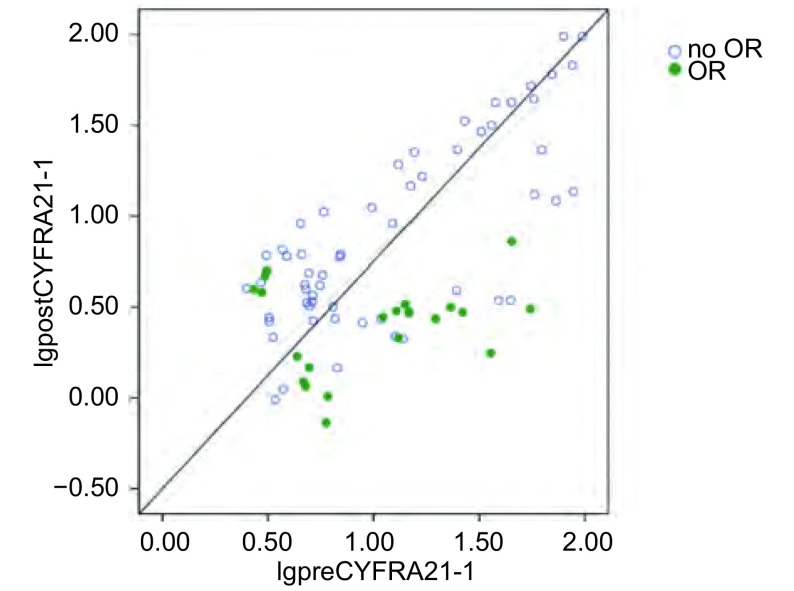
根据影像学OR的不同显示的化疗前和化疗2周期后血清CYFRA21-1的自然对数散点图（g/L，Mean±SD） Logarithm scatter diagram of pre-chemotherapy and post-chemotherapy CYFRA21-1 levels by imaging OR (g/L, Mean±SD)

### 2.4 影像学OR与血清CYFRA21-1水平下降的关系

应用ROC曲线评估化疗2周期后血清CYFRA21-1表达水平下降在预测化疗疗效能否达到影像学OR中的效能。血清CYFRA21-1下降≥60%时，血清CYFRA21-1的ROC曲线下面积为0.745（95%CI: 0.604-0.886），诊断OR的敏感性为81.0%，特异性为74.6%。根据ROC分析结果，将治疗后血清CYFRA21-1水平下降≥60%定义为血清CYFRA21-1反应，因为在这个水平上有最佳的诊断影像学OR的敏感性和特异性。在80例可评价病例中，32例（40.0%）出现血清CYFRA21-1反应，21例达到影像学OR的患者中17例（81.0%）出现血清CYFRA21-1反应，59例治疗无效的患者中仅15例（25.4%）出现血清CYFRA21-1反应（χ^2^=19.90, *P* < 0.001）。而年龄、性别、疾病分期、病理类型、PS评分、白细胞计数和CYFRA21-1基线水平与化疗能否达到影像学OR无统计学关联（表 2）。

**2:** 影响影像学OR的单因素分析 Univariate analysis of related factors on imaging OR

Related factors	No. of patients (%)		*P*
	No OR (*n*=59)	OR (*n*=21)	
Age (year)			0.150
≤65	36	9	
>65	23	12	
Gender			0.758
Male	40	15	
Female	19	6	
Clinical stage			0.286
Ⅲ	23	11	
Ⅳ	36	10	
ECOG PS scores			0.948
0	37	13	
1-2	22	8	
Histology			0.062
Squamous cell carcinoma	16	11	
Adenocarcinoma	35	7	
WBC baseline level			0.597
≤10 000/mm^3^	22	6	
>10 000/mm^3^	37	15	
CYFRA21-1 response			< 0.001
Yes	15	17	
No	44	4	
CYFRA21-1 baseline level			< 0.001
Normal	14	15	
>3.2 ng/mL	45	6	

*Log istic*分析也进一步证实了血清CYFRA21-1反应与化疗后影像学OR的相关性（OR=12.467, 95%CI: 3.619-42.943, *P* < 0.001）。

### 2.5 血清CYFRA21-1反应与预后的关系

在研究期间，80例患者中有74例患者死亡。所有患者的中位生存期为9.9个月（2.6个月-26.3个月），不同预后因素对患者生存期的影响见表 3。

**3:** 不同预后因素对NSCLC患者生存期的影响（*n*=80） Influence of different prognostic factors on survival time of NSCLC patients (*n*=80)

Related factors	*n*	MST (month)	95%CI	*P*
Age (year)				0.4681
≤65	45	9.8	8.9-10.7	
>65	35	11.2	9.6-12.8	
Gender				0.091
Male	55	10.8	9.5-12.1	
Female	25	9.7	9.2-10.2	
Clinical stage				0.572
Ⅲ	30	10.0	9.3-10.7	
Ⅳ	50	10.2	9.0-11.4	
EOCG PS scores				0.001
0	50	11.1	10.0-12.2	
1-2	30	9.4	6.9-11.9	
Histology				0.233
Squamous cell carcinoma	27	10.2	8.7-12.6	
Adenocarcinoma	42	9.4	8.5-10.3	
WBC baseline level				0.588
≤10 000/mm^3^	28	10.2	7.7-12.7	
>10 000/mm^3^	52	10.0	9.1-10.9	
OR				0.012
Yes	21	12.2	11.5-12.9	
No	59	9.7	8.9-10.5	
CYFRA21-1 response				< 0.001
Yes	34	12.3	11.6-13.0	
No	50	8.9	7.3-10.5	
CYFRA21-1 baseline level				0.001
Normal	8	9.0	6.2-11.8	
>3.2 ng/mL	72	10.7	9.8-11.6	

单因素生存分析结果显示，CYFRA21-1基线水平、CYFRA21-1反应、影像学OR及PS评分是影响患者预后生存期的重要预后因素，而年龄、性别、疾病分期及WBC计数则与预后无关。出现血清CYFRA21-1反应患者的生存期明显长于未出现血清CYFRA21-1反应者（12.3个月*vs* 8.9个月，*P* < 0.001），其生存曲线见图 2。

**2:**
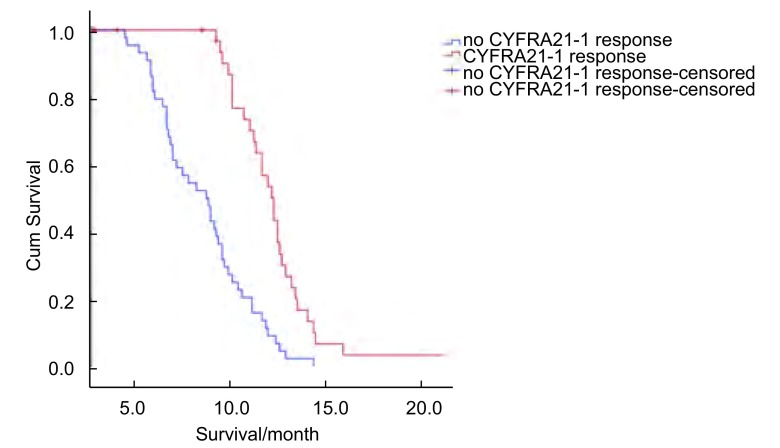
血清CYFRA21-1反应/无反应NSCLC患者的生存曲线 Survival curve of NSCLC patients with/without serum CYFRA21-1 response

*Cox*多因素分析结果证实，仅血清CYFRA21-1基线水平、血清CYFRA21-1反应、PS评分是影响预后的独立因素，而影像学是否达到OR与生存无关（表 4）。

**4:** 影响NSCLC患者生存时间相关预后因素的多因素分析 Multivariate survival analysis of related prognostic factors in NSCLC patients

Characteristic	HR	95%CI	*P*
ECOG PS scores			< 0.001
0	1.0		
1-2	2.929	1.720-4.988	
OR			0.238
No	1.0		
Yes	0.671	0.346-1.320	
CYFRA21-1 baseline level (ng/mL)			0.037
Normal	1.0		
Elevated, >3.2 ng/mL	0.374	0.148-0.944	
CYFRA21-1 response			0.001
No	1.0		
Yes	0.339	0.182-0.630	

### 2.6 血清CYFRA21-1反应与病理类型的关系

27例鳞癌患者中，24例化疗前CYFRA21-1水平高于正常阈值，化疗前和化疗2周期后血清CYFRA21-1中位值分别为8.88 ng/mL（2.71 ng/mL-55.49 ng/mL）和3.17 ng/mL（0.73 ng/mL-52.01 ng/mL），化疗后CYFRA21-1水平较化疗前明显下降（*P* < 0.000 1）；42例腺癌患者中，37例化疗前CYFRA21-1水平高于正常阈值，化疗前和化疗2周期后血清CYFRA21-1中位值分别为9.83 ng/mL（2.51 ng/mL-97.15 ng/mL）和5.01 ng/mL（0.98 ng/mL-97.71 ng/mL），化疗后CYFRA21-1水平较化疗前明显降低（*P*=0.001）。血清CYFRA21-1反应的鳞癌患者达到影响学OR的比例为62%，而血清CYFRA21-1无反应的鳞癌患者达到影响学OR的比例仅20%（*P*=0.025）；血清CYFRA21-1反应的腺癌患者达到影响学OR的比例为39%，血清CYFRA21-1无反应的腺癌患者达到影响学OR的比例为4%（*P*=0.003）。所有鳞癌患者的中位生存期为10.2个月（95%CI: 8.7-12.6），出现血清CYFRA21-1反应患者的中位生存期明显长于CYFRA21-1未反应者（12.3个月*vs* 9.5个月，*P*=0.016）；所有腺癌患者的中位生存期为9.4个月（95%CI:8.5-10.3），出现血清CYFRA21-1反应患者的中位生存期明显长于CYFRA21-1未反应者（12.3个月*vs* 7.7个月，*P* < 0.000 1）。出现血清CYFRA21-1反应的患者中鳞癌患者与腺癌患者中位生存期无明显差异（12.3个月*vs* 12.3个月，*P*=0.715）。

## 3 讨论

本研究是国内第一个根据化疗后CYFRA21-1水平下降判定进展期NSCLC患者化疗疗效和对预后生存期影响的研究。

由于依据影像学技术判定实体瘤疗效的RECIST标准不能准确判定不具有可测量病灶患者的疗效^[8]^，更未考虑影像学显示的肿块内肿瘤细胞是否存活，因此往往导致存活肿瘤组织被过高或过低的评价。如术前行新辅助化疗的患者，化疗后虽然患者临床症状及生活质量均有明显改善，手术切除的肿块中也有大量坏死的肿瘤组织，但其肿瘤体积缩小并不明显，甚至略有增大^[9]^，说明影像学检测的肿瘤体积大小的改变并不能反映化疗的真实疗效，因为有时治疗后肿瘤组织大部分均已坏死，其“存活肿瘤细胞”已明显减少或消失，但在影像学上其肿瘤的体积仍未变化或略有缩小^[10]^。因此，仅把根据影像学肿瘤体积大小的改变作为判定化疗是否有效的唯一标准，不能全面反映化疗的真实疗效。故而，人们开始寻找替代RECIST标准而进行疗效评价的新标准。

2008年美国肝病研究学会（American Association for the Study of Liver Disease, AASLD）对肝癌的RECIST标准进行了修订^[11]^，提出了“存活肿瘤”的概念，并将其定义为：动态CT或MRI时动脉期显示造影剂摄取的病灶。AASLD对RECIST的修订建议是以存活肿瘤为评估对象，排除了坏死肿瘤的干扰，不仅评估肿瘤体积的大小，还对肿瘤组织是否存活进行判断，同时弱化了肿瘤体积的改变在评估肿瘤客观疗效中的作用，使评估的疗效更具真实性。而作为判定NSCLC化疗疗效的RECIST标准，同样也遭到人们的质疑，人们开始寻找替代它的新的疗效评定标准。Sasaki等^[12]^应用PET-CT检查对肿瘤组织摄取^18^F-FDG水平进行检测，发现肿瘤组织摄取^18^F-FDG水平下降可以早期预测NSCLC化疗疗效。Hoekstra等^[13]^报道了应用^18^FDG PET在判定Ⅲa-N2期NSCLC患者化疗疗效方面优于CT，^18^FDG PET检测可以预测患者的生存期。虽然以代谢为基础的PET-CT利用放射性物质的摄取分辨肿瘤组织的代谢活性，同时又有CT评价肿瘤体积的变化，但由于这种方法价格昂贵，很难推广^[14]^。

肿瘤标志物是在肿瘤发生和增殖过程中由肿瘤细胞合成、释放或者是机体对肿瘤细胞反应而产生的一类物质，可反映肿瘤的存在和生长^[15]^。肿瘤标志物的检测是简易、经济、易推广的检查，且是不受主观因素影响的客观指标，因此常应用于肿瘤患者术后的随访，肿瘤的辅助诊断及治疗疗效的评估。如前列腺特异性抗原（prostate specific antigen, PSA）常用于前列腺癌疗效的评定^[16]^；CA125也常用于卵巢癌患者的疗效评定和预测预后^[17, 18]^；而CA199在评估转移性胰腺癌化疗疗效方面亦优于影像学评估法^[19]^。

尽管目前尚无一种敏感性、特异性均较高的NSCLC肿瘤标志物，但血清CYFRA21-1常用于评价NSCLC疗效和预后。Holdenrieder等^[20]^于2004年报道了CYFRA21-1水平可预测复发性NSCLC患者的化疗疗效，并优于影像学检测。2009年又报道了联合检测化疗前后核小体、CYFRA21-1、癌胚抗原（carcino-embryonic antigen, CEA）、神经元特异性烯醇化酶（neuron-specific enolase, NSE）及胃液素释放肽水平的变化与治疗反应密切相关，化疗后血清CYFRA21-1水平变化能预测疗效，并优于影像学技术^[21]^。血清CYFRA21-1下降不但可敏感地反映化疗引起的肿瘤缩小，并可预测进展期NSCLC患者的化疗疗效和生存期^[6, 22]^。

本研究及以往的报道共同质疑了影像学OR作为预后因素的有效性^[6, 23]^。Nisman等^[6]^研究了60例进展期NSCLC患者化疗2周期后血清CYFRA21-1变化水平与化疗疗效和预后的关系，发现影像学OR与生存期无明显统计学关联，而化疗2周期后血清CYFRA21-1变化水平却与影像学OR及生存期密切相关。Ardizzoni等^[23]^对107例进展期NSCLC进行了研究，以化疗2周期后血清CEA和CYFRA21-1下降≥20%定义为肿瘤标志物反应，肿瘤标志物反应与影像学OR和生存期密切相关，而影像学OR与生存无关，认为肿瘤标志物反应可作为影像学OR的替代指标。本研究结果显示，血清CYFRA21-1反应（CYFRA21-1下降≥60%）与影像学OR密切相关，其诊断OR的敏感性和特异性分别为81.0%和74.6%。这与既往多数研究结果相一致^[6, 20, 22, 23]^。由于血清CYFRA21-1反应可敏感地反映化疗引起的肿瘤体积的缩小，因此血清CYFRA21-1反应可作为理想的判定NSCLC患者化疗疗效（影像学OR）的替代指标。

本研究结果显示，化疗后血清CYFRA21-1反应不但与影像学OR密切相关，而且与进展期NSCLC患者预后生存期密切相关，化疗后血清CYFRA21-1水平变化可较好地区分进展期NSCLC患者预后的差异。单因素和多因素分析均显示，血清CYFRA21-1反应是重要的独立预后因素，虽然单因素分析显示，影像学OR与预后相关，但多因素分析结果却显示，影像学OR与预后无关。化疗2周期后达到影像学OR者和未达到OR者的生存期亦无明显差异，而化疗2周期后CYFRA21-1下降≥60%者的生存期却明显长于CYFRA21-1下降 < 60%者，说明CYFRA21-1反应较影像学OR对预后的影响更大。血清CYFRA21-1下降≥ 60%者有54.8%达到影像学OR，其中位生存期长达12.3个月，而CYFRA21-1下降 < 60%者仅有8.3%达到影像学OR，其中位生存期仅8.9个月，二者有明显的统计学差异。值得注意的是，达到影像学OR而未出现CYFRA21-1反应的可能性很小（仅3.6%），因此，对于化疗2个周期后仍未出现CYFRA21-1反应者，即使未行影像学评价也应及时停止化疗，因为此时如果继续化疗，患者达到影像学OR和生存获益的可能性极小。由此可见，根据化疗后血清肿瘤标志物水平的改变制定临床治疗决策是合理的。此外，除血清CYFRA21-1反应是影响进展期NSCLC患者的独立预后因素外，*Cox*多因素分析还显示，PS评分和CYFRA21-1基线水平也是影响预后的独立因素。

综上所述，影像学OR是反映肿瘤体积缩小的有效指标，但却与预后生存时间无关，血清CYFRA21-1反应不但可敏感地反映化疗引起的肿瘤体积的缩小，并可预测进展期NSCLC患者的预后生存时间，因此，血清CYFRA21-1反应可能是评价进展期NSCLC患者化疗疗效的较可靠指标。但其在应用于临床前，还需进一步进行扩大样本的前瞻性临床试验进行验证。
